# New Insights into the Molecular Interplay between Human Herpesviruses and Alzheimer’s Disease—A Narrative Review

**DOI:** 10.3390/brainsci12081010

**Published:** 2022-07-30

**Authors:** Evita Athanasiou, Antonios N. Gargalionis, Cleo Anastassopoulou, Athanassios Tsakris, Fotini Boufidou

**Affiliations:** 1 Department of Biopathology, Primary Healthfund, Bank of Greece, 105 64 Athens, Greece; evitathan@yahoo.gr; 2 Department of Biopathology, Eginition Hospital, Medical School, National and Kapodistrian University of Athens, 115 28 Athens, Greece; 3 Department of Microbiology, Medical School, National and Kapodistrian University of Athens, 115 27 Athens, Greece; cleoa@med.uoa.gr (C.A.); atsakris@med.uoa.gr (A.T.); 4 Neurochemistry and Biological Markers Unit, 1st Department of Neurology, Eginition Hospital, Medical School, National and Kapodistrian University of Athens, 115 28 Athens, Greece

**Keywords:** Alzheimer’s disease viral hypothesis, human herpesviruses, amyloid beta (Aβ), tau protein, neuroinflammation

## Abstract

Human herpesviruses (HHVs) have been implicated as possible risk factors in Alzheimer’s disease (AD) pathogenesis. Persistent lifelong HHVs infections may directly or indirectly contribute to the generation of AD hallmarks: amyloid beta (Aβ) plaques, neurofibrillary tangles composed of hyperphosphorylated tau proteins, and synaptic loss. The present review focuses on summarizing current knowledge on the molecular mechanistic links between HHVs and AD that include processes involved in Aβ accumulation, tau protein hyperphosphorylation, autophagy, oxidative stress, and neuroinflammation. A PubMed search was performed to collect all the available research data regarding the above mentioned mechanistic links between HHVs and AD pathology. The vast majority of research articles referred to the different pathways exploited by Herpes Simplex Virus 1 that could lead to AD pathology, while a few studies highlighted the emerging role of HHV 6, cytomegalovirus, and Epstein–Barr Virus. The elucidation of such potential links may guide the development of novel diagnostics and therapeutics to counter this devastating neurological disorder that until now remains incurable.

## 1. Introduction

Alzheimer’s disease (AD) is an irreversible progressive neurodegenerative disorder and the most prevalent cause of dementia worldwide, accounting for an estimated 60% to 80% of cases [1]. According to the World Alzheimer Report 2018, about 50 million people are suffering from AD globally, and this number is predicted to reach 152 million by mid-century with a considerable socioeconomic impact. Clinically, AD is often diagnosed on the basis of memory dysfunction about recent events that evolve to involve impairment in cognitive, behavioral, and functional aspects of a patient’s life. However, AD is considered to start decades earlier before clinical symptoms occur; thus, a better understanding of modifiable (e.g., cerebrovascular diseases, hypertension, diabetes, obesity, dyslipidemia, smoking) and non-modifiable (age, genetics, family history) risk factors might delay or prevent AD onset, as well as introduction of new therapeutic targets [2,3,4,5]. Age 65 is the threshold age for the differential diagnosis of early- from late-onset AD. Late-onset AD is characterized as sporadic, with high prevalence and morbidity, reflecting complex interactions between environmental and genetic factors [6]. Among them, the apolipoprotein E (APOE) ε4 allele has been identified as a major susceptibility gene and the most ordinary genetic factor for late-onset AD [7].

The disease is named after the German psychiatrist and neuropathologist Alois Alzheimer, who first reported a severe medical condition of the cerebral cortex in the postmortem brain of his first patient that suffered from memory loss and behavioral changes, characterized by the histological features that are today associated with AD: a massive loss of neurons and the presence of amyloid neuritic plaques and neurofibrillary tangles [8]. Over a century later, the extracellular senile plaques formed by amyloid beta (Aβ), the intracellular neurofibrillary tangles composed of abnormally hyperphosphorylated tau proteins, and the synaptic loss remain the pathological hallmarks of AD [9,10]. Although the mechanisms related to the disease pathology have been under substantial investigation and several hypotheses have been proposed, there is no accepted theory at the moment for explaining AD pathogenesis [2,9]. Emerging evidence in recent years supports that a wide spectrum of infectious agents (viruses, bacteria, fungi, and protozoa) can cross the blood–brain barrier (BBB) and might play a triggering role in AD development [11,12,13]. Among them, herpesviruses represent the most studied family of neurotropic pathogens [14].

### Association between HHVs and AD

Herpesviruses are double stranded-DNA viruses with a unique four-layered structure: a core containing the linear genome enclosed in an icosahedral capsid that is surrounded by an amorphous protein coat, the tegument, and by a lipid envelope bearing membrane-associated proteins [15]. Human herpesviruses (HHVs) are distributed worldwide, and more than 90% of adults are infected by one or multiple HHVs [16]. Based on their biological properties, HHVs are classified into three sub-families: the alpha (α), beta (β), and gamma (γ). Herpes simplex virus (HSV) 1, 2 and varicella zoster virus (VZV) belong to the α sub-family; cytomegalovirus (CMV) and HHV 6 and 7 are β-herpesviruses, whereas Epstein–Barr virus (EBV) and Kaposi sarcoma-associated herpesvirus (KSHV) are members of the γ sub-family. HHVs enter the nervous system by hematogenous or neuronal spread depending on the mechanism and location of exposure and can cause a neurologic disease during primary infection or following reactivation from a state of lifelong latency [17]. 

Almost three decades ago, different research groups demonstrated the presence of HSV 1 DNA in the postmortem brain of both AD patients and elderly people without AD [18,19,20]. In 1997, Itzhaki and colleagues first provided strong evidence for a linkage between HSV 1 infection and AD, supporting that the reactivation of HSV 1 in the central nervous system is more harmful in AD patients who carry the APOE-ε4 allele and that the combination of these two factors increases the likelihood of disease [21]. APOE-ε4 allele has been reported to promote brain infiltration by HSV 1, to enhance the attachment and the entry of HSV 1 into the host cells, as well as to increase the viral load in the brain [22,23]. The latter appears to have a detrimental effect on Aβ accumulation, further influencing the Aβ fibril formation and oligomeric Aβ stabilization that is essential for accelerating the early seeding of Aβ pathology [22,24]. It is also noteworthy that APOE facilitates lipid antigen presentation by CD1+ antigen presenting cells to naïve natural killer T (NKT) cells [25,26]. NKT cells have a critical role in the modulation of inflammatory responses that could lead to neuronal damage, and research has been recently conducted to shed light to NKT cells’ involvement in AD [27]. On the other hand, NKT cells are recognized as a mechanism of the innate immunity against viral infections. However, it is revealed that HSV 1 downregulates CD1 from infected human primary dendritic cells suppressing NKT cell activation as an immune evasion strategy [28].

In 2005, Wozniak and colleagues found a greater localization of HSV-DNA in Aβ brains compared with the aged-matched non-affected controls, supporting the hypothesis that virus directly impacts the pathological hallmarks of AD [29]. Readhead et al. recently published massive molecular and bioinformatics analyses of postmortem brain samples from four independent cohorts of AD patients, reporting the increased presence of herpesviruses in AD brains versus matched controls [27]. Although HSV 1 is the most thoroughly studied microbe in the context of AD pathogenesis, other herpesviruses have also been under investigation for possible implication, including CMV [30,31,32], EBV [33,34,35], HHV 6, and HHV 7 [33,36]. However, it should be mentioned that most of these viruses were found in the brain in a relatively low proportion of AD patients and elderly controls, compared to HSV 1, apart from HHV 6, which was detected in 70% of AD patients and 40% of age-matched controls, respectively [37]. The considerable overlap of HHV 6 and HSV 1 in the brain suggests that the two HHVs might act together in the development of AD. It is well established that HSV 1 is responsible for the induction of the major features of AD, while the contribution of the other HVVs is suspected, but it remains to be confirmed [37,38]. Since no effective vaccine has yet been developed to prevent HSV infection in humans, HSV 1 therapy is based on antiviral drugs such as acyclovir, penciclovir, foscarnet, and BAY 57-1293 which target virus replication [39]. Several studies have demonstrated that the above drugs and other anti-herpetic compounds greatly reduce tau phosphorylation and Aβ formation driven by HSV 1 [40,41,42,43]. Thus, anti-herpetic molecules could be used as potent treatment agents to inhibit or moderate AD progression, especially in patients with a documented history of recurrent HSV 1 infection [44,45]. Consequently, different clinical trials are ongoing towards this direction [44], and these attempts are expected to add considerable information regarding the linkage between HHVs and AD.

Apart from HHVs, other neurotropic viruses are reported to have an impact on cognitive decline within the context of AD, including the human immunodeficiency virus (HIV), the human T cell leukemia virus type I (HTLV-1), the influenza virus, and the severe acute respiratory syndrome coronavirus 2 (SARS-CoV-2) [11,12,46]. Moreover, current findings support that patients with chronic hepatitis C virus (HCV) infection appear to exhibit cognitive impairment and that it may increase the risk for dementia [47,48,49]. However, the mechanisms by which HCV infection increases the risk of dementia remain to be clarified [50].

Different molecular mechanisms have been proposed to explain how HHVs promote AD development, such as mechanisms involved in extracellular Aβ deposition, tau protein hyperphosphorylation, autophagy, neuroinflammation, oxidative stress, and apoptosis. This review focuses on these molecular mechanistic links between HHVs and AD that could introduce potential new targets for preventing or treating the disease that until now remains incurable.

## 2. Methodology

A PubMed search was conducted using specific keywords in order to identify, at first, the articles that point out which HHVs are related to AD development and then the articles that describe the mechanisms by which HHV infections may factor into AD pathology. Emphasis was mainly given on recent experimental studies that illustrated molecular mechanisms involved in Aβ deposition, tau protein hyperphosphorylation, autophagy, oxidative stress, apoptosis, and neuroinflammation. The keywords used for the literature search in different combinations are mentioned below: dementia, AD, HHVs, HSV 1, HSV 2, EBV, CMV, HHV 6, HHV 7, Aβ, tau protein, autophagy, oxidative stress, apoptosis, inflammation, synaptic loss, neuronal death. The retrieved relevant articles were systematically reviewed and critically analyzed.

## 3. Aβ Accumulation and Tau Hyperphosphorylation in AD

Aβ peptides are formed within the amyloidogenic pathway through the successive cleavage of a neuronal trans-membrane glycoprotein, the amyloid precursor protein (APP), by membrane-bound proteolytic enzymes [2]. Firstly, the extracellular domain of APP is cleaved by β-secretase, resulting in the production of the soluble N-terminal fragment and the intramembranous C-terminal fragment. The latter is subsequently subjected to cleavage by γ-secretase to release the Aβ peptide and a cytoplasmic polypeptide named APP intracellular domain (AICD). At low concentrations, Aβ is considered to serve a number of physiological functions, including neurogenesis, neuronal growth and survival, protection against oxidative stress, regulation of synaptic activity and plasticity, maintenance of BBB structural integrity, and surveillance against infections [51,52]. On the other hand, the imbalance between Aβ production and clearance leads to Aβ monomer accumulation, which due to its sequence has a high tendency to aggregate-forming toxic oligomers [2].

Under physiological conditions, the microtubule-associated protein tau is basically expressed in neurons in the brain and peripheral nerves where it is mainly located in the cytoplasm of axons, with much lower amounts in somatodendritic compartments [53]. It is essential for the stability, assembly, and dynamics of neuronal microtubules, and the microtubules are important for cytoskeleton structure and activity to serve the trafficking of different vesicles and organelles [54]. In addition, tau contributes to several physiological processes including neurogenesis and synaptogenesis, synaptic functions in terms of learning and memory, neuronal excitability, myelination, motor function, iron homeostasis, and DNA protection and chromosomal stability [55]. In the brain, tau is subjected to various posttranslational modifications, and the role of these modifications in protein function remains unknown [56]. Among them, phosphorylation is the most studied. The hyperphosphorylation of tau protein leads to conformational changes and then to the formation of aggregates (paired helical filaments and/or neurofibrillary tangles), which are associated with microtubule destabilization, synaptic loss, and neurodegeneration [57].

### HHVs Infections’ Association with Aβ Accumulation and Tau Hyperphosphorylation

**Figure 1:**
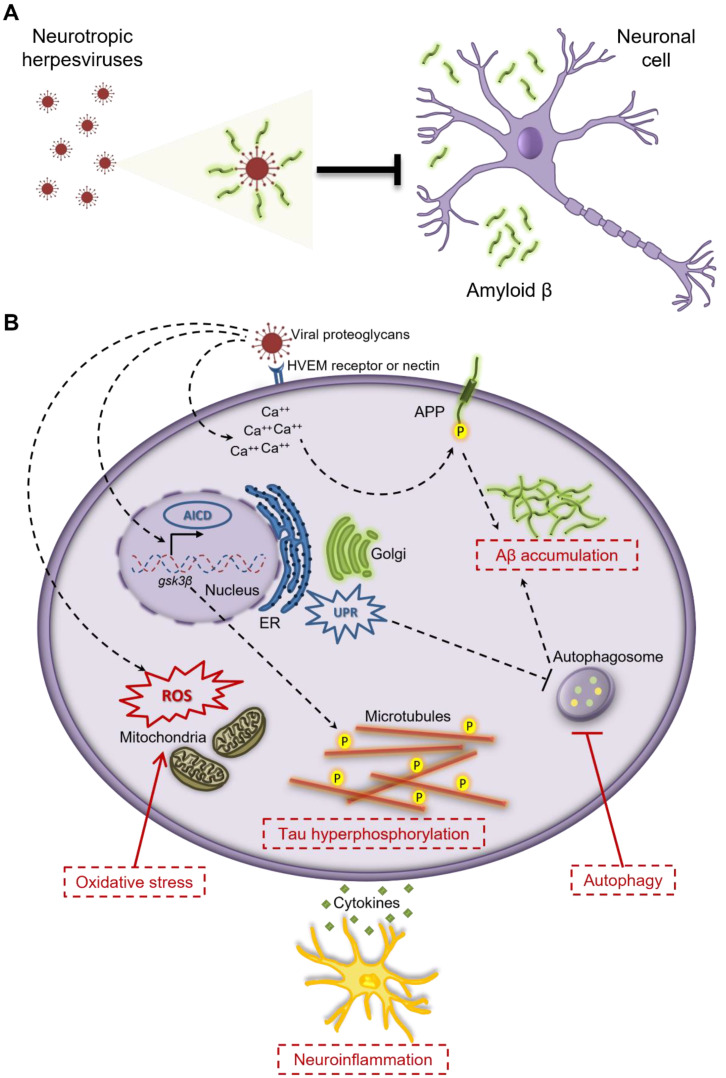
Neurotropic human herpesviruses drive the generation of AD hallmarks directly via interactions with the viral surface (**A**), or indirectly by affecting different molecular mechanisms (**B**), such as mechanisms involved in Aβ deposition, tau protein hyperphosphorylation, autophagy, oxidative stress, and neuroinflammation. HVEM: Herpesvirus Entry Mediator, AICD: APP Intracellular Domain, *gsk3β*: glycogen synthase kinase 3β, UPR: Unfolded Protein Response, APP: Amyloid Precursor Protein, Aβ: Amyloid β, ER: Endoplasmic Reticulum, ROS: Reactive Oxygen Species.

Mounting evidence demonstrates that HHVs can drive Aβ deposition and tau phosphorylation directly via interactions with the viral surface or specific viral proteins or indirectly by affecting upstream molecular pathways (Figure 1). 

The term amyloid is generally used to describe a conformational state, where proteins are transformed into insoluble aggregates of fibrillar morphology, and several biological or non-biological surfaces have been proven that are able to lower the energy barrier to nucleation promoting amyloid aggregation via a catalytic pathway named heterogeneous nucleation mechanism [58]. This mechanism is currently considered a critical factor regarding amyloid depositions induced by viruses. Aβ was found to act directly on HSV 1 by binding to its surface glycoproteins in a cell-free system preventing viral entry into fibroblast, epithelial, and neuronal cells, and thus inhibiting HSV 1 replication [59]. Eimer and colleagues also revealed that the Aβ attachment to HHV surface glycoproteins accelerates Aβ deposition and results in protective viral entrapment activity in 5XFAD mouse and 3D human neural cell culture infection models against HSV 1, HHV 6A, and HHV 6B [60]. Likewise, Ezzat and colleagues found that HSV 1 catalyzes the amyloid formation of Aβ in vitro, and a higher tendency of virus-mediated amyloid catalysis is observed for the more amyloidogenic Aβ1-42 peptide compared with the shorter, less amyloidogenic Aβ1-40 peptide [61]. There is a structural, dynamical difference between the two isoforms of Aβ. The two extra residues of Aβ1-42 confer a β-sheet character in its C-terminus that could explain the greater aggregation propensity of this isoform [62,63,64]. It is well established that β-sheets have been implicated in the formation of the fibrils and protein aggregates observed in amyloidosis [65]. In the same study, a significant increase in Aβ1-42 accumulation was noticed in the hippocampi and cortices of 5XFAD mice intracranially injected with HSV 1 in comparison with the non-infected animals. Taken together, the above findings indicate that Aβ peptides could be considered a novel class of antimicrobial agent against neurotropic infections caused by HHVs, but their overproduction may contribute to amyloid plaque formation and AD progression.

Furthermore, it was shown that HSV 1 infection promotes a striking increase in the intracellular levels of Aβ in cultured neuronal and glial cells [66,67,68,69]. Piacentini and colleagues demonstrated that HSV 1 binding to the neuronal cell membrane provokes a complex of electrophysiological responses that lead to significantly elevated intracellular calcium levels [67]. These signals trigger the phosphorylation of threonine at position 668 of APP, and thus, APP is subjected to multiple cleavages by secretases and caspases, resulting in Aβ accumulation. Moreover, HSV 1 infections seem to alter APP metabolism. HSV 1 activates the double-stranded (ds) RNA-activated protein kinase (PKR) that catalyzes the phosphorylation of the eukaryotic initiation factor 2-alpha (eIF2-α), a GTP-binding protein that in turn activates β-secretase translation, leading to Aβ production [70]. Research data also revealed that intracellular HSV 1 particles undergo frequent dynamic interplay with APP in a manner that facilitates viral transport and alters APP subcellular distribution and transport, upregulating its amyloidogenic processing [71]. In more details, HSV 1 capsids and APP-containing membranes co-localize and travel together through the cytoplasm at fast-anterograde transport rates. As a result, APP is mis-localized in HSV 1 infected cells, and then, it may be subjected to phosphorylation and proteolysis [69,71]. During HSV 1 infection, AICD accumulates within the nucleus of infected neuronal cells and binds to the promoter region of nep gene that encodes neprilysin (NEP), increasing its transcription [72]. NEP is a zinc metallopeptidase that has the capability of degrading monomeric and oligomeric forms of Aβ and plays a pivotal role in the maintenance of Aβ homeostasis in the brain [73]. NEP function as an Aβ-degrading enzyme introduces a protective mechanism against amyloid plaque formation in AD pathology. However, in a recent study, intranasal delivery of NEP in a transgenic AD mouse model rapidly eliminated Aβ plaques, but later on, this elimination caused a dramatic compensation of plaques in the cortex [74]. The authors proposed that NEP degrades the large Aβ plaques in the brain, which then are seen as smaller Aβ plaques, or that the transgenic mice activate a compensatory process to produce more Aβ plaques. MiR-H1, a miRNA encoded by HSV 1 and mainly expressed in productive infection, was found to target ubiquitin protein ligase E3 component n-recognin 1 (Ubr1), a RING-type E3 ubiquitin ligase that causes the degradation of proteins bearing “destabilizing” N-terminal residues, such as Aβ [75]. Ubr1 silencing mediated by miR-H1 reveals a novel molecular mechanism by which HSV 1 boosts Aβ gathering. The HHV 6A infection of microglial cells also promoted Aβ accumulation, as well as tau phosphorylation [76]. Recently, HHV 6A U4 protein was found to interact with cullin-RING E3 ubiquitin-protein ligases [77], a highly polymorphic E3 collection composed of a cullin backbone onto which carriers of activated ubiquitin and a diverse assortment of adaptors are bound to recruit appropriate substrates for ubiquitylation [78]. The above research findings led to the hypothesis that HHV 6A U4 protein competes with APP for binding to E3 ubiquitin ligase, and thus it prevents APP proteasomal degradation leading to increased APP expression and Aβ deposition.

In 2009, Wozniak and colleagues elucidated the mechanism involved in tau phosphorylation caused by HSV 1 infection, demonstrating that HSV 1 induces the activation of two enzymes, the glycogen synthase kinase 3β (GSK3β) and the protein kinase A (PKA) [79]. In this study, GSK3β proved to be responsible for tau phosphorylation at serine 202, threonine 212, serine 396, and serine 404, while tau phosphorylation at serine 214 occurred via the activity of PKA. Previous research data revealed that the viral US3 protein kinase interacts with and activates host PKA, further mimicking its functions [80]. In accordance with the above findings, AICD accumulated during HSV 1 infection within the nucleus of neuronal cells binds to the promoter region of gsk3β gene, enhancing its transcription [72]. Furthermore, neuroblastoma cells infected by HSV 1 generate increased levels of phosphorylated tau proteins that show nuclear localization at sites of viral DNA replication, whereas cyclin-dependent kinase (cdk) inhibitors reverse the above effect speculating that HSV 1 could contribute to tau phosphorylation through the deregulation of cdk activity [81]. Like HSV 1, the infection of human neuroblastoma cells with HSV 2 induces the main neurodegeneration markers associated with AD, including tau hyperphosphorylation, abnormalities in APP proteolytic processing resulting in intracellular Aβ accumulation, and impairment of autophagy [82].

## 4. Autophagy Impairment Caused by HHVs Infections

Autophagy is a catabolic process through which cells remove toxic components such as protein aggregates, misfolded proteins, and damaged organelles ensuring the maintenance of homeostasis and the adaptation to stressful conditions. This mechanism is highly important for the survival and homeostasis of post-mitotic cells, such as neurons that cannot dilute the unwanted cellular components by cell division. Autophagy is involved in the metabolism of Aβ [83] and tau proteins [84]. This fact, together with the observation that HHVs may dysregulate or impair the autophagic pathways of the host in order to survive, suggests another mechanistic link between infection by HHVs and AD pathology (Figure 1).

A recent study demonstrated for the first time that HHV 6A infection reduces autophagy in astrocytoma cells and primary neurons and activates unfolded protein response (UPR), a highly specific signaling pathway in the endoplasmic reticulum (ER) that recognizes the accumulation of unfolded or misfolded proteins [85]. According to the research results, autophagy reduction could be attributed to the altered lysosomal acidification caused by HHV 6A infection and could be considered a mechanism through which the virus increases Aβ production. The interplay between autophagy and UPR activation is of high importance, since the latter may promote the autophagic process in order to alleviate cells from ER stress. In the same study, UPR activation results in an increase of tau protein phosphorylation at multiple serine residues (such as 202, 396, and 404), which may be induced by the activation of the ER stress sensor protein PERK (Protein Kinase RNA-like ER Kinase) that also acts by activating GSK3β [86,87].

HSV 1 infection also appears to exert a modifying influence on the autophagic process [88,89]. Beclin 1 is an adaptor multi-domain protein with a large interactome that is subjected to different post-translational modifications and plays a key role in the synthesis and maturation of autophagosomes, whereas it may regulate autophagy in either a positive or a negative manner [90]. The HSV 1 ICP34.5 (Infected Cell Protein 34.5) neurovirulence protein was found to directly interact with Beclin 1 in mammalian cells, antagonizing and then impeding the host autophagy response [91]. In the same study, it was demonstrated that a mutant HSV 1 lacking the ICP34.5 domain that binds to Beclin 1 fails to impair autophagy in neurons and to cause lethal encephalitis in mice. Like ICP34.5, HSV 1 Us11 protein also displays anti-autophagic activity via its direct interaction with PKR probably by blocking its activity [92]. Human neuroblastoma cells infected by HSV 1 exhibit an increased formation of microtubule-associated protein 1 light chain 3-II (LC3-II)-positive autophagic vesicles in which intracellular Aβ is localized, as well as an ineffective fusion between autophagosomes and lysosomes, indicating that this infection cause functional impairments in the late stages of autophagy [68,93]. 

Autophagy modulation has recently emerged as an effective strategy in the fight of neurodegenerative disorders, including AD [7,94,95,96]. Two different approaches have been under investigation towards this direction: the use of small molecule therapeutics (such as berberine, sirolimus, or trehalose) and the genetic intervention (i.e., gene therapy targeting TFEB or BECN1). Both approaches exhibited promising outcomes in both in vitro and in vivo models of AD [97,98,99,100,101], whereas drug repositioning studies are ongoing to reveal new autophagy modulators [94]. Several potential therapeutic strategies have also been proposed that target the ER stress signaling to combat AD pathology [102]. The use of pharmacological modulators of UPR, such as inhibitors of the integrated stress response, chemical chaperones, and anti-inflammatory drugs demonstrated promising results ameliorating cognitive deficits in AD mouse models [103,104,105,106].

It is worthwhile to mention that autophagy also occurs in oxidative stress [107], a process that reflects the significant imbalance in the equilibrium between the antioxidant mechanisms and the generation of reactive oxygen species (ROS) and reactive nitrogen species (RNS). Numerous studies support that oxidative stress contributes to AD pathogenesis and progression [108,109], whereas different viruses are commonly associated with the presence of oxidative stress in infected cells [110,111]. HSV 1 infection was found to increase ROS levels in mouse P19N neural cells as a prerequisite for viral replication [112]. ROS production was also induced in mouse microglial cells infected by HSV 1 via the viral stimulation of toll-like receptor (TLR) 2, resulting in lipid peroxidation and neurotoxicity [113]. Santana and colleagues revealed that oxidative stress enhances the autophagic flux impairment that is provoked by HSV 1 infection in neuronal cell models [114]. In line with the above findings, a recent study combining experimental data and a functional whole genome analysis based on microarrays demonstrated that oxidative stress and HSV 1 infection enhance the lysosomal content, inhibit the activity of lysosomal enzymes, and impair the endocytosis-mediated degradation of the epidermal growth factor receptor (EGFR) in a cell model of neurodegeneration [115]. In more details, it was shown that the activity of cathepsins B, K, S, and D/E was significantly reduced in this model. Cathepsins are proteases with serine, cysteine, or aspartic acid residues, which are activated in the acidic environment of lysosomes and are vital for a wide range of physiological functions including autophagy [116]. Lysosome alkalization caused by oxidative stress as a response to HSV 1 infection may impair cathepsins activity. The inactivation of cysteine cathepsins B and L was found to affect the proper lysosomal function (as it was assessed by a decreased EGFR degradation and the accumulation of several lysosomal proteins), and then to get involved in the generation of amyloidogenic products (Aβ, C-terminal fragment, and β-secretase) that are derived from the APP cleavage [117]. Currently, combined biochemical and redox proteomic approaches revealed that HSV 1 reactivations trigger oxidative modifications of proteins linked to AD pathogenesis in the cortex of infected mice [118]. In this context, the oxidation of the glucose-regulated protein 78 (GRP78) leads to its dysfunction and consequent aberrant UPR activation, while the oxidation of collapsin response-mediated protein 2 (CRMP2) was found preserved. CRMP2 is a multifunctional adaptor protein in the central nervous system able to bind and stabilize microtubules playing a key role in cytoskeletal dynamics, vesicle trafficking, and synaptic physiology [119]. Upon oxidation on Cys504, a disulphide-linked CRMP2 homodimer forms a transient complex with thirodoxin1, leading to the activation of GSK3β [120], which is involved in tau phosphorylation. 

A recent systematic study based on the analysis of transcriptome datasets from seropositive or seronegative patients for CMV, EBV or HHV 6, and AD or Parkinson’s disease patients revealed that infected by HHVs patients share common molecular signatures with AD patients. In more detail, host response against the abovementioned HHVs seems to have an impact in oxidative stress mechanisms involved in AD pathology through the activation of sirtuin-1 and peroxisome proliferator-activated receptor-gamma coactivator (PGC)-1alpha pathway [121]. Sirtuin-1 has been shown to mediate the deacetylation of PGC-1alpha, resulting in the upregulation of its transcriptional function, and the overexpression of PGC1α leads to reduced Aβ generation, particularly by regulating the expression of β-secretase [122].

However, oxidative stress does not represent a HHVs-specific phenomenon with an impact on AD pathology. Neuron cells are particularly vulnerable to oxidative stress due to their high polyunsaturated fatty acid content in membranes, high oxygen consumption, and weak antioxidant defense [123]. Environmental stress, nutritional factors (e.g., redox-active metals), and inflammation may increase the oxidative stress, leading to higher Aβ production [124,125,126]. Elderly individuals are more prone to oxidative stress, which partially accounts for AD susceptibility in aging populations [127,128].

## 5. Neuroinflammation Induced by HHVs Infections

Taking into account that the brain is no longer considered an immune-privileged organ and that patients with AD demonstrate elevated levels of inflammatory markers and AD risk genes related to innate immune functions, neuroinflammation has emerged as a major driving factor in neurodegeneration and AD pathology besides the classical hallmarks (Figure 1) [129]. The inflammatory response in AD brain is characterized by the activation of astrocytes and microglial cells that are thought to be important elements of both early and late AD pathogenesis [130,131,132]. Although a number of studies have demonstrated that glial activation prevents AD progression by facilitating Aβ clearance in the brain, a growing body of evidence supports that the exacerbated or hindered activation of glial cells enhances the production of proinflammatory cytokines and Aβ in the brain, triggering a cascade of events that eventually lead to neurodegeneration and cognitive decline [130]. Thus, neuroinflammation could represent a promising drug target to avert AD progression.

Neurotropic viral infections may interplay with or exacerbate inflammatory processes in the central nervous system and then alter host cell immunity, contributing to AD development [133]. In this context, HSV 1 is again the most investigated virus among HHVs. It was shown that human trigeminal ganglia latently infected by HSV 1 exhibit persisting infiltration by lymphocytes and increased expression of cytokines that affect viral replication (interferon (IFN)-γ and tumor necrosis factor (TNF)-α), chemokines that attract immune cells (IFN-γ-inducible protein 10 (IP-10)), and the C-C motif chemokine ligand 5 (CCL5 or RANTES), [134]. These findings also highlighted the fact that HSV 1 latency is accompanied by a chronic inflammatory process but without any neuronal destruction. Interestingly, the infection of human primary neural cells with HSV 1 induced the upregulation of a brain-enriched miRNA (miRNA-146a) that is associated with proinflammatory signaling in stressed brain cells and AD, suggesting a possible role in viral evasion from the complement system and the activation of key elements of the arachidonic acid cascade known to contribute to Alzheimer-type neuropathological change [135,136].

Microglial cells represent the first line of defense against HSV 1 by releasing pro-inflammatory cytokines and chemokines [137], whereas they are considered the major cellular source of inducible nitric oxide synthase (iNOS) facilitating the production of nitric oxide (NO) [138]. Enhanced NO synthesis is an important contributor to oxidative stress-associated neurodegeneration, while NO is reported to create some sort of a cycle triggering Aβ deposition, which in turn activates resident glial cells that secrete NO; this continuous cycle is thought to have a detrimental impact on AD patients [139]. NO also aggravates the toxic effect of glutamate via an ionotropic receptor in microglia, the N-methyl-D-aspartate (NMDA) receptor [140]. Moreover, HSV 1 infection was found to prompt murine microglia to generate ROS through Toll-like receptor (TLR) 2, which exacerbated inflammation leading to neural oxidative damage [113,141]. In response to toxicity, damaged neurons can generate damage associated molecular patterns (DAMPs) and other microglia activators, including matrix metalloproteinase-3 (MMP3), a-synuclein and melanin, and further interfere with AD pathogenesis [137]. Following HSV 1 infection, microglia are the main producers of type I IFNs among cells in the central nervous system via the cyclic GMP-AMP synthetase (cGAS) and stimulator of IFN genes (STING) pathways [97,142], and it is well known that type I IFN response drives neuroinflammation and synapse loss in AD [143,144].

It is worthy to mention that, as an AD pathological feature, neuroinflammation is not restricted to HHVs. Other neurotropic viruses provoke inflammatory responses that may have an impact on AD development. SARS-CoV-2 is a highly neuroinvasive neurotropic virus that invades cells through angiotensin-converting enzyme 2 (ACE2) receptor-driven pathway. Upon the activation of microglia by SARS-CoV-2, cytokines such as IL-1 and TNF activate astrocytes, which in turn can produce inflammatory molecules, including TNF-α, ROS, and NO [145,146]. Furthermore, neurotropic RNA viruses are able to induce inflammatory events after viral entry that may prime the central nervous system to develop neurodegenerative disorders [147].

There is accumulating evidence that myelin damage is an important part of the pathological changes observed in AD, and may even precede Aβ and tau pathologies [148]. Although oligodendrocytes are the main cells that orchestrate myelination, astrocytes and microglia also contribute to this process and could play either beneficial or detrimental roles by promoting or impairing the endogenous capability of oligodendrocyte progenitor cells to promote spontaneous re-myelination after myelin loss [149]. The myelin basic protein is the major structural component of myelin and has been proven to bind Aβ and inhibit Aβ fibril formation, possibly regulating Aβ1-42 deposition and then amyloid plaques formation in the brains of AD patients [150,151]. On the other hand, Aβ induces oligodendrocytes death by activating the sphingomyelinase–ceramide pathway and hinders myelin formation [152]. Thus, myelin loss and decreased levels of myelin basic protein may accelerate Aβ deposition and Aβ plaque formation in AD patients [148]. In a recent study, it was demonstrated that HSV 1 infection generates brain inflammation and multifocal demyelination in the cotton rat *Sigmodon hispidus*, while the process of re-myelination that rapidly follows demyelination leads to the formation of partially re-myelinated plaques [153].

Although a few studies have been conducted regarding the role of EBV in AD pathogenesis compared to HSV 1, it is well established that during its latency and reactivation phases EBV may generate a systemic immune response to stress, which promotes inflammation associated with cognitive decline during aging [33,35]. Under normal circumstances, peripheral lymphocytes cross the BBB to patrol the brain parenchyma. EBV may infect peripheral blood mononuclear cells and use them to enter the brain via the “Trojan horse” mechanism [154]. In a recent study, B lymphocytes isolated from an AD patient were immortalized upon infection with EBV and then co-cultured with neuronal cells in order to construct a cellular model to mimic the normal conditions of the bloodstream [155]. B lymphocytes were able to produce high levels of the inflammatory cytokine TNF-α that in turn promoted Aβ accumulation and tau protein hyperphosphorylation [155,156]. Gate and colleagues suggested that the adaptive immunity might also play a significant role in disease progression based on the discovery of CD8+ T effector memory CD45RA+ (TEMRA) cells with proinflammatory and cytotoxic functions in AD patients and the identification of two EBV antigens that triggered this immune response, the Epstein–Barr nuclear antigen 3 (EBNA3A) and the trans-activator protein BZLF1 [34,157,158]. In another attempt to provide insights into the mechanistic role of EBV in AD, almost a hundred EBV proteins were analyzed for their aggregation proclivity using an in silico analysis [159]. BNLF-2a is the tail-anchored protein encoded by EBV, which inserts ER after translation and blocks the transporter protein (TAP) associated with antigen processing, thereby providing immune escape properties to infected cells [160]. BNLF-2a can further promote AD progression by inhibiting TAP and down-regulating major human histocompatibility complex (MHC)-I and II expression, leading to the accumulation of neuronal cells and viral polypeptides in the environment [159].

## 6. Concluding Remarks

During their long-lasting stay in the human brain, HHVs have several opportunities to interfere with practically every mechanism that has been proposed to underlie AD pathophysiology (Table 1). Strong evidence supports that infections by HHVs trigger molecular events associated with autophagy, oxidative stress, and neuroinflammation with a profound effect on AD pathology. Interestingly, there is an interplay between the aforementioned biological processes, since they may induce and/or enhance each other. HSV 1 and HHV 6A have been reported to dysregulate autophagy in neuronal cells with an impact on Aβ accumulation and tau protein metabolism. HSV 1 impairs autophagy through its neurovirulence proteins: ICP34.5 [91] and US11 [92], whereas HHV 6A dysregulates the autophagic process via the activation of UPR [85]. Accumulating evidence also suggests that HHVs infection results in the increased production of oxidant species, like they do infections by other neurotropic viruses such as HIV [161]. Oxidative stress induced by HSV 1 modifies lysosomes by increasing their content, decreasing the activity of the lysosomal enzymes, or impairing cathepsins that are involved in the generation of amyloidogenic products. CMV, EBV, and HHV 6 have been reported that they also have an impact on oxidative stress via the activation of the sirtuin-1 and PCG-1alpha pathway, which influences Aβ generation [121,122]. Furthermore, it is well established that the autophagy reduction and the increased production of oxidant species promote inflammation. The release of inflammatory agents stimulates Aβ production in astrocytes, while soluble tau oligomers may be secreted into the extracellular environment and contribute, independently or combined with Aβ, to synaptic dysfunction [162]. Glial cells play either a protective or restorative role in neurons, and thus impairment of their function by HHVs infection could contribute to AD progression. Although there is a great number of molecules derived from HSV 1, HHV 6A, EBV, and CMV that are identified to play a key role in AD pathology, there is a long way to pave in order to elucidate all the molecular mechanisms involved, to examine the possible etiology of other HHVs or to investigate the potent contribution of other factors such as gut microbiota. Shedding more light on the molecular pathways involved in the aforementioned biological processes could lead to new therapeutic targets in order to prevent or hinder the neurodegeneration that leads to AD advancement.

The aim of this review was to highlight potential mechanistic links between HHVs and AD and, thus, to help identify possible new treatment targets. Given the series of failure over the past two decades on disease-modifying treatments and the controversial approval of Aducanumab by the Food and Drug Administration (FDA) in 2021, in conjunction with the multifactorial nature of the disease, strategies aiming at the elimination of molecules that could serve as putative triggers, if not causative factors, for AD is a reasonable approach. This possibility is supported by an observational retrospective cohort study on electronic health databases in Taiwan that provided evidence for a lower incidence of dementia in HSV-infected patients who had received an antiviral drug compared to non-treated subjects [45]. Currently, there are two ongoing Phase II placebo–control clinical trials of valacyclovir in patients with mild AD and HSV seropositivity (NCT03282916) and in mild cognitive impairment patients exhibiting AD biomarkers (NCT04710030). These studies are expected to shed light on the HSV involvement in AD progression and pathogenesis.

Additionally, over the last five years, there is increasing evidence for an altered folding of several host encoded cellular proteins associated with their self-aggregation into disease specific pathological lesions within the brain [163]. Besides this, evidence for the transmissibility of unusual pathogenic Aβ and tau isoforms has given rise to the “prion hypothesis” for AD and the conceptualization of a double prion disorder [164]. Under this perspective, the augmentation of abnormal protein folding by HHVs and other environmental factors in AD and the specific molecular mechanistic links involved glares as an intriguing new horizon.

## Figures and Tables

**Table 1:** HHVs proteins and their targets in neuronal cells within the context of AD pathology.

HHV Type	Viral Molecule	Host Cell Target	Biological Process	Reference
HSV 1	MiR-H1	Urb1	Aβ accumulation	[75]
	US3 protein kinase	PKA	Tau phosphorylation	[80]
	ICP34.5 protein	Beclin 1	Autophagy blockage	[91]
	Us11 protein	PKR	Autophagy blockage	[92]
		MiRNA-146a	Neuroinflammation	[135,136]
HHV 6A	U4 protein	APP	Aβ accumulation & tau phosphorylation	[77]
HHV 6 EBV CMV		Sirtuin-1	Oxidative stress	[121] [122]
EBV	EBNA3A, BZLF1	TEMRA cells	Neuroinflammation	[157]
	BNLF-2a	TAP	Immune evasion	[159]

Abbreviations: HHVs; Human Herpesviruses, AD; Alzheimer’s Disease, HSV 1; Human Simplex Virus 1, EBV; Epstein–Barr Virus, CMV; Cytomegalovirus, MiR-H1; microRNA-H1, ICP34.5; Infected cell protein 34.5, EBNA3A; Epstein–Barr nuclear antigen 3, TEMRA; CD8+ T effector memory CD45RA+, TAP; Transporter protein associated with antigen processing, Urb1; ubiquitin protein ligase E3 component n-recognin 1, PKA; protein kinase A, PKR; double-stranded (ds) RNA-activated protein kinase, APP; Amyloid Precursor Protein, Aβ; amyloid β.

## Data Availability

Not applicable.
